# Performance of Plasma Amyloid β, Total Tau, and Neurofilament Light Chain in the Identification of Probable Alzheimer's Disease in South China

**DOI:** 10.3389/fnagi.2021.749649

**Published:** 2021-10-27

**Authors:** Bin Jiao, Hui Liu, Lina Guo, Xinxin Liao, Yafang Zhou, Ling Weng, Xuewen Xiao, Lu Zhou, Xin Wang, Yaling Jiang, Qijie Yang, Yuan Zhu, Lin Zhou, Weiwei Zhang, Junling Wang, Xinxiang Yan, Beisha Tang, Lu Shen

**Affiliations:** ^1^Department of Neurology, Xiangya Hospital, Central South University, Changsha, China; ^2^National Clinical Research Center for Geriatric Disorders, Central South University, Changsha, China; ^3^Key Laboratory of Hunan Province in Neurodegenerative Disorders, Central South University, Changsha, China; ^4^Department of Geriatrics, Xiangya Hospital, Central South University, Changsha, China; ^5^Department of Radiology, Xiangya Hospital, Central South University, Changsha, China; ^6^Key Laboratory of Organ Injury, Aging and Regenerative Medicine of Hunan Province, Changsha, China; ^7^Engineering Research Center of Hunan Province in Cognitive Impairment Disorders, Central South University, Changsha, China; ^8^Hunan International Scientific and Technological Cooperation Base of Neurodegenerative and Neurogenetic Diseases, Changsha, China

**Keywords:** Alzheimer's disease, plasma biomarkers, amyloid-beta, total-tau, neurofilament light chain

## Abstract

**Background:** Alzheimer's disease (AD) is the most common type of dementia and has no effective treatment to date. It is essential to develop a minimally invasive blood-based biomarker as a tool for screening the general population, but the efficacy remains controversial. This cross-sectional study aimed to evaluate the ability of plasma biomarkers, including amyloid β (Aβ), total tau (t-tau), and neurofilament light chain (NfL), to detect probable AD in the South Chinese population.

**Methods:** A total of 277 patients with a clinical diagnosis of probable AD and 153 healthy controls with normal cognitive function (CN) were enrolled in this study. The levels of plasma Aβ42, Aβ40, t-tau, and NfL were detected using ultra-sensitive immune-based assays (SIMOA). Lumbar puncture was conducted in 89 patients with AD to detect Aβ42, Aβ40, t-tau, and phosphorylated (p)-tau levels in the cerebrospinal fluid (CSF) and to evaluate the consistency between plasma and CSF biomarkers through correlation analysis. Finally, the diagnostic value of plasma biomarkers was further assessed by constructing a receiver operating characteristic (ROC) curve.

**Results:** After adjusting for age, sex, and the apolipoprotein E (*APOE*) alleles, compared to the CN group, the plasma t-tau, and NfL were significantly increased in the AD group (*p* < 0.01, Bonferroni correction). Correlation analysis showed that only the plasma t-tau level was positively correlated with the CSF t-tau levels (*r* = 0.319, *p* = 0.003). The diagnostic model combining plasma t-tau and NfL levels, and age, sex, and *APOE* alleles, showed the best performance for the identification of probable AD [area under the curve (AUC) = 0.89, sensitivity = 82.31%, specificity = 83.66%].

**Conclusion:** Blood biomarkers can effectively distinguish patients with probable AD from controls and may be a non-invasive and efficient method for AD pre-screening.

## Introduction

Currently, ~50 million people in the world are living with dementia and every 3 s a new case of dementia is diagnosed (Christina, 2018). Alzheimer's disease (AD) is the most common neurodegenerative dementia in older people and is characterized by progressive cognitive decline and behavioral defects with a complex and heterogeneous pathophysiology.

A preclinical phase of ≥20 years may occur before the clinical diagnosis of AD, during which no or only subtle symptoms appear, adding to the difficulty of early diagnosis and prevention (Jack et al., 2013; Jansen et al., 2015). At present, the most well-established AD biomarkers are mainly based on the core pathological features, including amyloid β (Aβ) deposition [detected by cerebrospinal fluid (CSF)] Aβ42 levels, amyloid positron emission tomography (PET), neurodegeneration [CSF total tau (t-tau)] and phosphorylated (p)-tau levels, structural MRI, and hypometabolism on fluorodeoxyglucose (FDG)-PET (Desikan et al., 2009; Mattsson et al., 2009; Landau et al., 2012). Nevertheless, high invasiveness, expensive costs, and limited availability hinder their clinical application. Therefore, noninvasive, cost-effective, and easily accessible biomarkers are desperately needed for the identification of AD.

To date, many studies have focused on blood-based biomarkers for the diagnosis of AD; somewhat promising results have mainly been achieved with Aβ, tau, and neurofilament light chain (NfL) in the blood. Previous studies have described the excellent performance of plasma Aβ-related peptides for the prediction of AD using different methods (Chen et al., 2019; Vergallo et al., 2019). The plasma t-tau level is a promising candidate marker because it is a brain-specific protein that is mainly expressed in central nervous system (CNS) neurons, indicates neuronal damage, and is derived from the brain parenchyma and transported to the CSF and blood (Schraen-Maschke et al., 2008). It has also been shown to enhance the prediction of dementia and is suggested to serve as a biomarker for risk stratification in dementia prevention trials (Mielke et al., 2017). NfL, the main component of the axonal cytoskeleton, is mainly expressed in large-caliber myelinated axons and released into the CSF following neuroaxonal injury (Petzold, 2005). Recently, robust studies have shown that NfL level in the peripheral blood is a promising biomarker for tracking neurodegenerative changes in patients with AD and increased levels are related to brain atrophy, brain hypometabolism, and decreased cognitive function (Zetterberg et al., 2016; Mattsson et al., 2017; Mayeli et al., 2019). Due to the poor disease specificity, the clinical application in detecting AD is limited (Wilke et al., 2016; Bridel et al., 2019). Compelling and emerging evidence has highlighted the potential of plasma p-tau181 and p-tau217, both of which have shown exceptional sensitivity and specificity to widespread AD pathology at autopsy and in patients with underlying AD pathology confirmed by other biomarkers (Janelidze et al., 2020; Karikari et al., 2020; Lantero Rodriguez et al., 2020; Palmqvist et al., 2020; Thijssen et al., 2020). However, due to technical limitations, these results are predominantly based on the Caucasian populations, and studies on blood biomarkers in Chinese populations are hard to achieve and still lacking. To our knowledge, only one study has detected plasma p-tau level in a Chinese population, which mainly focused on the correlation between p-tau181 and cognitive function (Xiao et al., 2021). Further studies are warranted to explore these biomarkers in currently underrepresented populations. Considering the heterogeneity and complexity of AD etiology, it is difficult to use a single biomarker to reflect the comprehensive pathological changes and disease diagnosis. Few domestic studies have integrated plasma Aβ, tau, and NfL simultaneously to evaluate their comprehensive diagnostic efficacy for AD and assess their consistency with classic CSF core biomarkers.

Therefore, in this cross-sectional study, we simultaneously detected the levels of classic biomarkers in the plasma of patients with AD and cognitively normal (CN) individuals from South China, including Aβ42, Aβ40, t-tau, and NfL, assessed their performance in discriminating patients with probable AD from CN participants to evaluating their ability to diagnose AD.

## Materials and Methods

### Participants

A total of 430 individuals, including 277 patients with probable AD and 153 CN participants, were enrolled from the Department of Neurology, Xiangya Hospital, Central South University, between March 2017 and December 2019. The inclusion criteria for patients were as follows: (1) memory complaints from the patient or guardian; (2) ability to cooperate with physical examination and neuropsychological tests; (3) brain atrophy confirmed by CT or MRI, and (4) diagnosis of probable AD by two or more experienced neurologists from Xiangya Hospital according to the criteria of the National Institute on Aging and Alzheimer's Association (NIA-AA) (McKhann et al., 2011). In this study, the inclusion and exclusion criteria for controls were as follows: (1) no subjective cognitive complaints and no objective impairment in cognitive tests, including the Clinical Dementia Rating Scale (CDR) (CDR scores = 0) and Mini-Mental State Examination (MMSE; combined with educational attainment, illiteracy > 17, primary school > 20, and junior high school and above > 24); (2) no brain organic or functional disease; (3) no hypertension, diabetes, hyperhomocysteinemia, or other systemic diseases; and (4) matched with patients with probable AD by age and sex.

The study protocol was approved by the Institutional Review Board of Xiangya Hospital of Central South University in China. Written informed consent was obtained from each participant or guardian.

### Neuropsychological and Cognitive Assessment

Participants in the AD group underwent a battery of neuropsychological tests, including the MMSE, Montreal Cognitive Assessment (MoCA), Activities of Daily Living (ADL), and Neuropsychiatric Inventory (NPI). The MMSE was also administered to the CN group.

Further, all participants were interviewed by two neurologists specializing in neurodegenerative disease, and the severity of cognitive impairment was assessed using the CDR.

### The Apolipoprotein E (*APOE*) Genotyping

Venous blood was collected from all participants in tubes containing ethylenediaminetetraacetic acid (EDTA). Genomic DNA was extracted using the standard phenol-chloroform extraction method. All DNA samples were diluted to 50 ng/μl. A 581-bp fragment was amplified using the following primers: forward 5′-CCTACAAATCGGAACTGG-3′ and reverse 5′-CTCGAACCAGCTCTTGAG-3′. PCR was performed as previously described (Jiao et al., 2014). Each PCR product was sequenced using an ABI 3730xl DNA analyzer (ABI, Louis, MO, USA).

### CSF Collection and Analysis

The cerebrospinal fluid was obtained from lumbar puncture samples. In this study, as lumbar puncture was carried out on a voluntary basis, a total of 188 patients refused to undergo the procedure, and 89 patients were tested for CSF core biomarkers. Briefly, participants were placed in the left lateral position for lumbar puncture. The L3–L5 intervertebral spaces were selected as the puncture site. The CSF samples were processed within 2 h after the standard lumbar puncture, and hemorrhagic samples were excluded (Teunissen et al., 2009). Each sample was centrifuged at 2,000 × *g* for 10 min, and the CSF samples were separated and stored in an enzyme-free microcentrifuge tube at −80°C. All samples were subjected to a maximum of two freeze-thaw cycles. All analyses of CSF core AD biomarkers (Aβ42, Aβ40, t-tau, and p-tau) were measured using enzyme-linked immunosorbent assay (ELISA) and performed by experienced technicians in strict accordance with the instructions of the manufacturer within 1 week of sample collection. Briefly, samples were added to the reagent wells, and the plate was incubated for 3 h at 22°C ± 2°C. After washing, horseradish peroxidase solution was added and incubated for 90 min at 22°C ± 2°C. The plate was then washed with the provided washing buffer, and substrate solution was added. After 30 min incubation protected from light, stop-solution was added, and the optical density (OD) was measured using a microplate reader (Thermo, Waltham, MA, USA), at 450 nm, corrected by the reference OD at 620 nm within 30 min of adding the stop solution. Two technical replicates are performed on samples and standards, and the average value of the replicates is used for statistical analysis. All measurements were performed in a blinded manner. Specifically, Aβ42 level in CSF <651 pg/ml, or Aβ42/Aβ40 ratio ≤ 0.1, is defined as positive amyloidosis (corresponding to A+ in the ATN framework); p-tau > 61 pg/ml in CSF is defined as neurofibrillary tangles (corresponding to T+ in the ATN framework); t-tau ≥ 290 pg/ml in CSF is defined as nerve cell death (corresponding to A+ in the β amyloid deposition, pathologic tau, and neurodegeneration [ATN] framework).

### Plasma Protein Quantification

Venous blood was collected in tubes containing EDTA and centrifuged at 2,000 × *g* for 10 min at 4°C. The obtained plasma was divided into ~500 μl aliquots and frozen at −80°C. All samples underwent no more than three freeze-thaw cycles (Keshavan et al., 2018). The samples were rapidly thawed at 22 ± 2°C and then centrifuged at 10,000 × *g* prior to analysis to prevent any sample debris from interfering with the measurements. Plasma Aβ42, Aβ40, t-tau, and NfL concentrations were measured simultaneously using the single-molecule array (SIMOA)-HD1 platform (SIMOA; Quanterix, Billerica, MA, USA), which employed an automated SIMOA principle. Briefly, Aβ42, Aβ40, and t-tau levels were measured using a multiplex array (Neurology 3-Plex A Advantage Kit, N3PA), and NfL levels were measured using a single-analyte array (NF-light). The samples were measured using a two-step immunoassay. All analytical procedures were performed according to the protocol of the manufacturer by well-trained technicians who were blinded to the state of participant and clinical data, according to the protocol of the manufacturer. Samples with coefficients of variance (CV) of >20% were excluded from the analyses. In this study, the within-batch CV of all samples was <5%.

### Statistical Analysis

The normality of the distribution of the variables was assessed. Categorical data were analyzed using the χ^2^ test. Continuous variables were compared between two independent samples using the *t*-test or the Mann-Whitney *U* test, and differences between multiple independent samples were compared using the Kruskal-Wallis H test. Partial correlation analyses were performed to assess the correlations among plasma biomarkers, demographic characteristics, and clinical data. Diagnostic accuracy was evaluated using receiver operating characteristic (ROC) curve analysis and with logistic regression models. The area under the curve (AUC) and representative optimal sensitivity and specificity were used to evaluate the performance of the models. The statistical significance of the difference in AUCs between two different models was analyzed using Delong's test.

All tests were two-tailed, and *p* < 0.05 was considered statistically significant. All analyses were performed using SPSS version 24 (IBM, Armonk, NY, USA) and R (version 4.1.0). Data were visualized using Prism 8 software (GraphPad, San Diego, CA, USA).

## Results

### Demographic Characteristics

The demographic characteristics of the patients with probable AD and CN participants are summarized in Table 1, including 277 patients with probable AD and 153 CN participants. The mean age at onset (AAO) was 62.23 years, and the mean disease course was approximately 2.91 years. The sex ratio (F/M) of patients with probable AD was 172/105, which matched the CN group (99/54) (*p* > 0.05). In parallel, 160 (57.8%) patients with AD carried at least one *APOE4* allele. There was no significant difference in age at diagnosis (means the age at the time of blood extraction) between patients with probable AD and CN participants, but the educational levels were significantly different between the two groups (*p* < 0.001).

**Table 1:** Demographic and clinical characteristics of patients with probable AD and healthy controls.

	**AD (*n* = 277)**	**Control (*n* = 153)**	***p***
**Demographic and clinical characteristics**			
Age at diagnosis (Mean ± SD)	65.11 ± 10.57	64.5 ± 8.2	0.650
Age at onset (Mean ± SD)	62.23 ± 10.7	–	–
Sex (F/M, *n*)	172/105	99/54	0.591
Education level, years (Mean ± SD)	7.31 ± 4.38	8.99 ± 3.52	<0.001^*^
*APOE*4 (+/–, *n*)	160/117	26/127	<0.001^*^
Family history (+/–, *n*)	90/187	–	–
Disease course, years (Mean ± SD)	2.91 ± 2.13	–	–
MMSE (Mean ± SD)	12.0 ± 6.44	27.7 ± 2.3	<0.001^*^
MoCA (Mean ± SD)	7.63 ± 5.48	–	–
ADL (Mean ± SD)	36.79 ± 11.78	–	–
NPI (Mean ± SD)	19.20 ± 14.96	–	–
CDR (*n*, %)			
CDR = 0	0	153 (100)	–
CDR = 1	156 (56.3%)	0	–
CDR = 2	108 (39.0%)	0	–
CDR = 3	13 (4.7%)	0	–
**Plasma biomarkers (mean** **±** **SD)**			
Aβ42 (pg/ml)	14.18 ± 3.96	14.58 ± 3.33	0.02 (0.308)
Aβ40 (pg/ml)	275.77 ± 63.25	258.48 ± 50.36	0.002 (0.062)
Aβ42/Aβ40	0.052 ± 0.014	0.057 ± 0.013	<0.001^*^ (0.056)
t-tau (pg/ml)	4.12 ± 1.25	3.23 ± 1.12	<0.001^*^(<0.001^#^)
NfL (pg/ml)	28.76 ± 30.34	14.13 ± 10.25	<0.001^*^ (<0.001^#^)

*Demographic and clinical characteristics of patients with probable AD and healthy controls. Continuous variable comparisons between two independent samples were conducted via t-test or the Mann-Whitney U test. Categorical data were analyzed using the χ^2^ test. The p-value in the bracket refers to the p-value after adjusting for age (means the age at diagnosis), sex, and APOE alleles. The differences of plasma biomarkers between the AD and CN groups were performed correction for multiple comparisons*.

* *The difference between the groups is statistically significant (p < 0.05)*.

# *The difference between the groups is statistically significant (p < 0.01, Bonferroni corrected)*.

*AD, Alzheimer's disease; ADL, Activities of Daily Living; CDR, Clinical Dementia Rating Scale; MMSE, Mini-Mental State Examination; MoCA, Montreal Cognitive Assessment; NPI, Neuropsychiatric Inventory; NfL, neurofilament light chain; t-tau, total tau*.

### Differences in Plasma Biomarkers Levels

**Figure 1:**
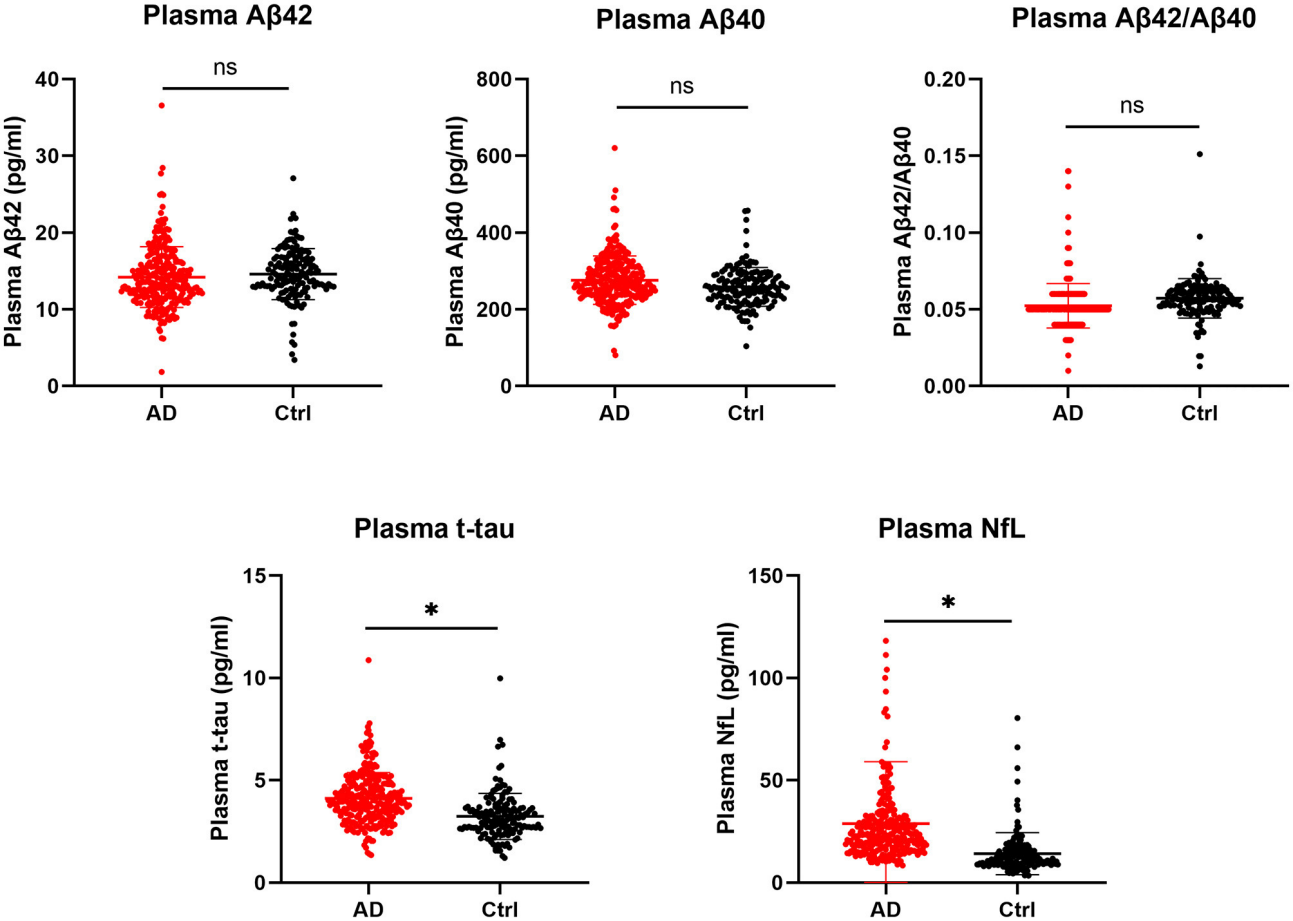
Comparison of plasma biomarkers between the AD and CN groups. Plasma biomarkers are presented as the means ± SD. The significance of differences between groups was determined using the Mann-Whitney *U* test. AD, Alzheimer's disease; CN, cognitively normal; t-tau, total tau; NfL, neurofilament light chain; ns, not significant; *p < 0.05.

Before correcting for confounding factors, all detected plasma biomarkers, including Aβ42, Aβ40, Aβ42/Aβ40, t-tau, and NfL, significantly differed between the AD and CN groups. Among them, the plasma Aβ42 and Aβ42/40 levels were significantly lower in the AD group than in the CN group (*p* = 0.02 and *p* < 0.001, respectively). In parallel, the levels of Aβ42/Aβ40, t-tau, and NfL showed an increasing tendency in patients with probable AD (*p* < 0.05; Table 1). However, after adjusting for age, sex, and *APOE* alleles, and Bonferroni correction, compared with the CN group, plasma t-tau, and NfL were significantly increased in the AD group (*p* < 0.001), whereas other plasma biomarkers, including Aβ42 and Aβ40, showed no significant difference (*p* = 0.308 and *p* = 0.062, respectively), Aβ42/Aβ40 showed a decreasing tendency, but the difference was not significant (*p* = 0.056; Table 1; Figure 1).

### Correlations Between Plasma Biomarkers and Demographic Characteristics

Next, we analyzed the correlations between plasma biomarkers and demographic data, including AAO, age at diagnosis, disease course, and education level. Associations between the levels of plasma biomarkers and demographic data were examined using partial correlation analyses with adjustment for age at diagnosis, sex, and *APOE* alleles. In addition, we compared the differences in plasma biomarkers according to sex, family history, and *APOE4* alleles distribution (Table 2). After adjustment for age at diagnosis, sex, and *APOE* alleles, Aβ42, Aβ40, Aβ42/Aβ40, and t-tau were not significantly associated with AAO in the probable AD group (*p* > 0.05), but NfL showed a significant association (*r* = −0.183, *p* < 0.001), which indicated that the earlier the AAO, the higher the level of NfL in plasma. In contrast, there was a significant positive correlation between age at diagnosis and the plasma NfL level (*r* = 0.235, *p* < 0.001). Meanwhile, plasma NfL was positively correlated with the disease course (*r* = 0.199, *p* < 0.001). Moreover, compared with patients with a positive family history of dementia, both plasma Aβ42 and Aβ42/Aβ40 were significantly lower in patients without a positive family history of dementia. Regarding *APOE* alleles, the Aβ42/Aβ40 ratio, and NfL were significantly lower in patients carrying the *APOE4* alleles than in non-carriers (*p* = 0.033 and *p* = 0.034, respectively), whereas there were no significant differences in other plasma biomarkers between the two subgroups (Table 2).

**Table 2:** Correlations of plasma biomarkers with demographics and neuropsychological assessments in patients with AD.

**CSF biomarkers**	**Plasma Aβ42**		**Plasma Aβ40**		**Plasma Aβ42/Aβ40**		**Plasma t-tau**		**Plasma NfL**	
	***r***	***p***	***r***	***p***	***R***	***p***	***r***	***p***	***r***	***p***
Age at onset	−0.041	0.503	−0.018	0.763	−0.007	0.907	−0.025	0.682	−0.198	<0.001^*^
Age at diagnosis	0.040	0.513	0.081	0.184	−0.048	0.426	0.011	0.861	0.235	<0.001^*^
Sex (F/M)	–	0.285	–	0.097	–	0.619	–	0.425	–	0.077
Education level (years)	−0.024	0.691	−0.039	0.518	0.025	0.684	0.058	0.336	0.105	0.083
Disease course (years)	0.041	0.497	0.018	0.765	0.007	0.912	0.021	0.729	**0.199**	<0.001^*^
Family history (+/–)	–	<0.001^*^	–	0.627	–	<0.001^*^	–	0.630	–	0.218
*APOE*4 (+/–)	–	0.349	–	0.465	–	0.033^*^	–	0.666	–	0.034^*^
MMSE	0.054	0.375	0.078	0.198	−0.023	0.701	−0.079	0.191	−0.044	0.474
MoCA	0.069	0.258	0.085	0.160	−0.015	0.811	−0.059	0.334	0.008	0.891
ADL	−0.115	0.058	−0.090	0.139	−0.006	0.920	−0.014	0.818	−0.035	0.566
NPI	−0.051	0.402	0.021	0.731	−0.071	0.242	0.012	0.845	0.021	0.729

*Correlations of plasma biomarkers with demographics and neuropsychological assessments in patients with AD. Correlations between plasma biomarkers and AAO were assessed by partial correlation with age at diagnosis, sex, and APOE alleles adjustment. Correlations between plasma biomarkers and age at diagnosis were assessed by partial correlation with AAO, sexes, and APOE alleles adjustment. Correlations between plasma biomarkers and educational level were assessed by partial correlation with age (means age at diagnosis), sexes, and APOE alleles adjustment. Correlations between plasma biomarkers and neurological assessment were assessed by partial correlation analyses with age at diagnosis, sex and APOE alleles, educational levels adjustment*.

* *The difference between the groups is statistically significant (p < 0.05)*.

*AD, Alzheimer's disease; ADL, Activities of Daily Living; MMSE, Mini-Mental State Examination; MoCA, Montreal Cognitive Assessment; NPI, Neuropsychiatric Inventory; NfL, neurofilament light chain; t-tau, total tau*.

### Correlations Between Plasma Biomarker Levels and Neuropsychological Assessments

We analyzed the correlations between plasma biomarkers and neuropsychological assessments, including MMSE, MoCA, ADL, and NPI (Table 2). Associations between the levels of plasma biomarkers and neuropsychological assessments were examined using partial correlation analyses with adjustment for age, sex, *APOE* alleles, and education level. The results showed that all the plasma biomarkers showed no significant association with neuropsychological assessments, including MMSE, MoCA, ADL, and NPI.

To further analyze the correlations between plasma biomarkers levels and disease severity, the patients with probable AD were divided into three subgroups according to the CDR score. No significant difference in plasma biomarkers was found among patients with different disease severity.

### Correlations Between Plasma and CSF Biomarkers

According to the ATN diagnosis framework, not all clinically diagnosed patients with probable AD were compatible with the biologically defined AD. Specifically, among 89 patients who underwent lumbar puncture, 70 (78.7%) patients were diagnosed with AD continuum (A + T + N +: 39 cases, A + T + N–: 8 cases, A + T – N +: 9 cases, A + T – N –: 14 cases). The correlations between plasma and CSF biomarker levels in patients with probable AD were determined to assess the efficacy of plasma biomarkers in reflecting changes in brain pathology, as shown in Table 3. After adjusting for age, sex, and *APOE* alleles, only plasma t-tau was positively correlated with t-tau in the CSF (*r* = 319, *p* = 0.003).

**Table 3:** Correlations between plasma biomarkers and CSF biomarkers in patients with AD (*n* = 89).

**CSF biomarkers**	**Plasma Aβ42**		**Plasma Aβ40**		**Plasma Aβ42/Aβ40**		**Plasma t-tau**		**Plasma NfL**	
	***r***	***p***	***r***	***p***	***r***	***p***	***R***	***p***	***r***	***p***
Aβ42	0.081	0.461	0.098	0.370	−0.011	0.923	−0.061	0.579	0.017	0.875
Aβ40	−0.081	0.458	−0.090	0.410	0.024	0.823	−0.158	0.146	−0.013	0.907
Aβ42/Aβ40	0.166	0.126	0.209	0.053	0.037	0.738	−0.081	0.456	0.180	0.097
p-tau	−0.150	0.169	−0.166	0.128	−0.002	0.982	0.065	0.551	−0.141	0.197
t-tau	−0.115	0.293	−0.151	0.164	−0.019	0.862	0.319	0.003^*^	−0.121	0.268

*Correlations between plasma biomarkers and CSF biomarkers in patients with AD (n = 89). Correlations between the levels of plasma biomarkers and CSF core biomarkers were assessed with partial correlation analyses with age, sexes, and APOE alleles adjustment. Age here refers to the age at diagnosis*.

* *The difference of the correlations between these biomarkers is statistically significant (p < 0.05)*.

*AD, Alzheimer's disease; NfL, neurofilament light chain; t-tau, total tau*.

### Diagnostic Performance of Plasma Biomarkers

**Figure 2:**
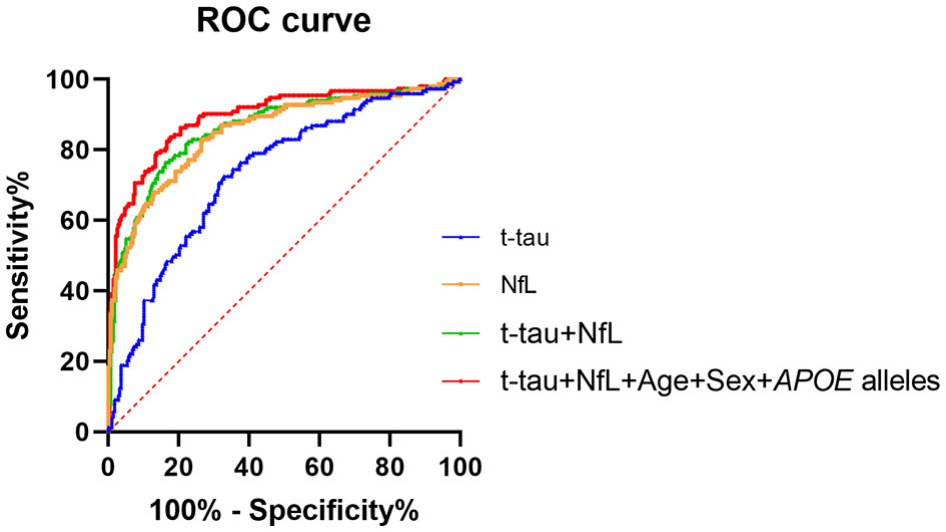
ROC curve analysis of plasma biomarkers for the AD diagnosis. NfL levels in plasma exhibited the best diagnostic efficacy as a single indicator (AUC = 0.85, sensitivity = 73.28%%, specificity = 83.00%). Combined with t-tau and NfL levels in plasma, the diagnostic performance was significantly improved (AUC = 0.86, sensitivity = 83.75%, specificity = 76.47%). The diagnostic model combining plasma t-tau and NfL levels, age, sex, and *APOE* alleles showed the best performance for the identification of probable AD (AUC = 0.89, sensitivity = 82.31%, specificity = 83.66%). Delong test was performed to compare the difference of different diagnostic models. AUC, area under the curve; ROC, receiver operating characteristic curve; NfL, neurofilament light chain; t-tau, total tau.

Finally, ROC curves were generated to evaluate the performance of the plasma biomarkers to discriminate patients with probable AD from controls (Figure 2). The cutoff value and its corresponding sensitivity and specificity were calculated using the maximum Youden index (Table 4). As a single plasma biomarker, NfL displayed the best diagnostic efficacy (AUC = 0.85, sensitivity = 73.28%, specificity = 83.00%). In addition, based on the results of differences in plasma biomarker levels, the model combining plasma t-tau and NfL showed the best diagnostic performance (AUC = 0.86, sensitivity = 83.75%, specificity = 76.47%). Furthermore, when age, sex, and *APOE* alleles were included in the combined model, it showed the best performance to distinguish probable AD from CN participants (AUC = 0.89, sensitivity = 82.31%, specificity = 83.66%), which was significantly better than other models (*p* < 0.05). In addition, we extracted 89 patients with AD who underwent CSF biomarker detection and analyzed the diagnostic performance of their plasma biomarkers through the ROC curve. The results showed that when plasma t-tau and NfL are included in the model, the diagnostic efficiency is better than that of any single plasma biomarker. After incorporating age, sex, and *APOE* alleles into the model, there was no statistical difference in the diagnostic performance of the two models, but the AUC of the latter reached the best (AUC = 0.89, sensitivity = 78.65%, specificity = 88.88%; Table 4).

**Table 4:** Performance of plasma biomarkers for probable AD diagnosis.

**Plasma biomarkers**	**Sensitivity (%)**	**Specificity (%)**	**AUC**	**95% CI**	***p***
t-tau	67.14 (66.29)	72.54 (78.43)	0.73 (0.74)	0.68–0.78 (0.67–0.80)	<0.001^*^ (<0.001^*^)
NfL	73.28 (77.52)	83.00 (82.35)	0.85 (0.86)	0.81–0.89 (0.78–0.90)	0.002^*^ (0.08)
t-tau+NfL	83.75 (87.64)	76.47 (73.20)	0.86 (0.86)	0.82–0.90 (0.81–0.90)	0.007^*^ (0.07)
t-tau + NfL + age + sex + *APOE* alleles	82.31 (78.65)	83.66 (88.88)	0.89 (0.89)	0.86–0.92 (0.86–0.94)	

*Performance of plasma biomarkers for probable AD diagnosis, including the sensitivity, specificity, AUC, and 95% CI. The values in brackets refer to the results of diagnostic efficacy analysis on a subset of patients who underwent lumbar puncture. The p-value is obtained by comparing each model with the combined model incorporating t-tau, NfL, age, sex, and APOE alleles through the Delong test. Age here refers to the age at diagnosis. AD, Alzheimer's disease; AUC, under the curve; CI, confidence interval; NfL, neurofilament light chain; t-tau, total tau*.

* *The difference of the correlations between these biomarkers is statistically significant (p < 0.05)*.

## Discussion

This study aimed to evaluate the ability of plasma biomarkers, including Aβ, t-tau, and NfL, to detect probable AD in a Chinese population. We first determined the differences in plasma biomarkers between patients with probable AD and CN participants. Second, a comprehensive model with high diagnostic efficacy of AD was constructed, including plasma t-tau, NfL, age, sex, and *APOE* alleles, which can be applied to perform preliminary screening in populations with a high risk of AD and may effectively reduce the application of lumbar puncture and PET examinations in clinical practice.

At present, a lot of efforts are devoted to comparing the difference of plasma Aβ between patients with AD and control, trying to diagnose AD through the less invasive and easily acceptable method. However, the results of different studies are still controversial. Studies adopting the SIMOA platform showed lower levels of plasma Aβ42 and Aβ42/Aβ40 in patients with AD than in controls (Janelidze et al., 2016; Li et al., 2019; Tosun et al., 2021). By contrast, some studies found that the levels of Aβ42 and Aβ42/Aβ40 were significantly increased in patients with mild cognitive impairment (MCI) or AD (Teunissen et al., 2018; Palmqvist et al., 2019). However, several studies showed that there were no significant differences in the plasma Aβ levels between patients with AD and controls (Hsu et al., 2017; Lövheim et al., 2017). The low abundance of Aβ in plasma and detection methods with different sensitivities, such as ELISA, SIMOA, immunomagnetic reduction (IMR), and Elecsys immunoassays, are the main reasons for these conflicting findings (Qu et al., 2021). To our knowledge, few domestic studies have evaluated these plasma biomarkers simultaneously in large cohorts from China or analyzed their correlations with typical AD biomarkers (Lue et al., 2017; Shi et al., 2019). In the present study, after adjusting for age, sex, and *APOE* alleles, the level of plasma Aβ42/Aβ40 showed a decreasing tendency but not a significant difference in patients with probable AD; meanwhile, the individual levels of plasma Aβ42 and Aβ40 were not significantly different from those of controls, which was consistent with the previous study (Olsson et al., 2016). Aβ40 is always used as a reference peptide to potentially explain the difference in CSF concentrations between individuals and the difference in sample preanalytical processing (Schauer et al., 2018). As for the correlation between plasma Aβ and neuropsychological assessment, previous studies have reported the association between plasma Aβ levels and cognitive performance (Hanon et al., 2018; Chen et al., 2019), which indicates that plasma Aβ can potentially reflect the cognitive function even monitor the progression of AD. In this study, after adjusting for confounding factors, there was no significant correlation between plasma Aβ and neuropsychological assessment.

Approximately 30–50% of blood Aβ is derived from a brain-to-blood transport mechanism, and a dynamic equilibrium exists between the peripheral blood and the CNS (Roberts et al., 2014). The levels of Aβ in the plasma reflect pathological changes in the brain to some extent. Robust studies have shown that plasma Aβ levels can reflect Aβ pathology in the brain using amyloid PET or CSF Aβ levels as the positive reference (Park et al., 2017; Hanon et al., 2018; Pérez-Grijalba et al., 2019; Schindler et al., 2019). Furthermore, the ratio in plasma appears to be associated with the increased risk of progression to AD dementia (Verberk et al., 2018). However, in this study, the associations of plasma and CSF Aβ were not significant. There are several possibilities to explain these results. First, in the present study, only 89 patients with probable AD were tested for CSF core biomarkers, and the small sample size of CSF cannot reflect the true relationship of Aβ between plasma and CSF. Second, in this study, the inclusion criteria of patients with AD were the NIA-AA criteria and not all patients met the biological definition of AD. Considering that the clinical symptoms of different types of neurodegenerative dementia have a high degree of overlap, there are several clinically diagnosed patients with probable AD without Alzheimer's pathology. Third, the different detection methods used for markers in CSF and plasma may also be one of the reasons for the weak correlation. Finally, in this study, the different disease severity of patients with AD may also explain part of the results.

In the present study, plasma t-tau levels were significantly higher in patients with probable AD than in healthy controls, similar to the findings of most published results (Mattsson et al., 2016; Lue et al., 2017), whereas several studies also found that plasma t-tau levels showed the opposite result or no significant difference between them (Sparks et al., 2012; Verberk et al., 2018; Qu et al., 2021). In a large meta-analysis, it was confirmed that patients with AD had higher plasma t-tau levels than controls, which may reflect neuronal damage as a nonspecific marker (Jack et al., 2018; Ding et al., 2021). Mattson et al. found that the higher plasma t-tau level was associated with AD dementia and showed significant correlations with poor cognition, greater atrophy, and hypometabolism during follow-up in the AD Neuroimaging Initiative study (Mattsson et al., 2016). In our study, after adjusting for age, sex, and *APOE* alleles, plasma t-tau was weakly correlated with CSF t-tau, which is consistent with previous studies that the association between plasma t-tau with CSF t-tau was weak or nonsignificant (Müller et al., 2017; Pase et al., 2019). Notably, accumulating evidence supports that plasma p-tau, the most promising biomarker for AD, shows outstanding performance in differential diagnosis, in relation to other biomarkers, neuropathology, prediction, progression monitoring, and prognosis (Mielke et al., 2018; Janelidze et al., 2020, 2021; Palmqvist et al., 2020). Due to the low concentration in the samples and limitations associated with the methodology, we did not measure p-tau levels in plasma. In our next study, we will increase the sample size to reevaluate whether plasma t-tau levels predict cognitive function and AD pathology and assess the utility of plasma p-tau levels for discriminating patients with AD from controls in the Chinese population.

Neurofilament light chain is a sensitive and promising biomarker for neurodegenerative disease, as it is released into CSF and plasma after axonal damage (Kuhle et al., 2015; Weston et al., 2017). Recently, Quiroz et al. found that plasma NfL levels increased with age and began to differentiate in *PSEN1* E280A mutation carriers from noncarriers as early as 22 years of age based on a large kindred study of patients with AD (Quiroz et al., 2020). Consistent with these results, the plasma NfL concentration was correlated with age and increased significantly as individuals aged in the present study. Meanwhile, the association between plasma NfL levels was positively correlated with disease course, which supports the hypothesis that the NfL is a sensitive marker of progressive myelinated axonal damage in the early stage of AD. However, it is not a specific indicator of the typical pathological changes associated with AD (Disanto et al., 2017). The correlation analysis showed that plasma NfL levels were not associated with AD core biomarkers in CSF, which is inconsistent with some previous study findings (Lewczuk et al., 2018; Mattsson et al., 2019). Combined with a recent longitudinal study showing that the plasma NfL level of participants who developed AD increased at a rate that was consistently higher than that of CN participants, and as early as 10 years before the clinical diagnosis of AD (de Wolf et al., 2020), these data indicate that it maybe a stable and useful biomarker for disease identification and for monitoring disease progression.

Notably, the results suggested that these plasma indicators were promising for discriminating patients with probable AD from CN participants. The peripheral biomarker panel as an initial screening strategy to identify people who should undergo further examinations, such as PET imaging or CSF testing, might be a critical step forward. Additionally, given the low concentrations of markers in plasma and the limited detection sensitivity, methodological limitations still exist. In this study, we unified the detection method by SIMOA technology to minimize possible experimental errors and promote the accurate detection of plasma biomarkers.

There are some limitations to this study. First, the study was a cross-sectional study without longitudinal follow-up to determine the differences in the trajectories of plasma biomarkers of patients in different AD stages and CN individuals. Second, due to the invasive characteristics of lumbar puncture, we failed to obtain the CSF data of all the participants in this study. Importantly, as the inclusion criteria for patients were probable AD but not biologically proven AD, there maybe several “AD” participants without Alzheimer's pathology as the primary cause of their symptoms, or controls with Alzheimer's pathology since AD is a continuum, where participants may present without any symptoms. Finally, patients with non-AD dementia were not recruited to compare differences in plasma biomarkers and verify the ability of the panel to distinguish patients with AD dementia from patients with other forms of dementia. Thus, head-to-head comparisons based on large-scale prospective studies that adopt unified and standardized inclusion criteria, detection, and analysis methods are necessary to assess the ability of plasma biomarkers for AD diagnosis in a Chinese population and their utility in monitoring pathological changes in the brain.

In summary, plasma biomarkers, including t-tau and NfL levels, were significantly different between the AD and CN groups after adjusting for age, sex, and *APOE* alleles. The diagnostic model that included t-tau, NfL levels, age, sex, and *APOE* alleles showed the best performance in discriminating patients with probable AD from CN participants. Although the accurate diagnosis of AD using plasma biomarkers is still challenging, the combination of multiple blood biomarkers can identify patients with probable AD from controls and is expected to serve as a potential method for pre-screening probable AD.

## Data Availability Statement

The raw data supporting the conclusions of this article will be made available by the authors, without undue reservation.

## Ethics Statement

The studies involving human participants were reviewed and approved by the Ethics Committee of Xiangya Hospital of the Central South University. The patients/participants provided their written informed consent to participate in this study.

## Author Contributions

BJ, HL, and LS: study design, acquisition of data, analysis and interpretation of data, and drafting/revising the manuscript. LG, XL, and YZho: analyzed the data and revised the manuscript for intellectual content. LW, XX, LuZ, XW, YJ, QY, YZhu, WZ, LiZ, JW, XY, and BT: data collection and analysis. All authors contributed to the article and approved the submitted version.

## Funding

This study was supported by the National Key R&D Program of China (Nos. 2020YFC2008500, 2017YFC0840100, and 2017YFC0840104 to LS, and No. 2018YFC1312003 to JW), the National Natural Science Foundation of China (Nos. 81671075 and 81971029 to LS, No. 82071216 to BJ, and No. 81901171 to XL), Hunan Innovative Province Construction Project (No. 2019SK2335 to BT), and the Youth Science Foundation of Xiangya Hospital (No. 2018Q020 to XL).

## Conflict of Interest

The authors declare that the research was conducted in the absence of any commercial or financial relationships that could be construed as a potential conflict of interest.

## Publisher's Note

All claims expressed in this article are solely those of the authors and do not necessarily represent those of their affiliated organizations, or those of the publisher, the editors and the reviewers. Any product that may be evaluated in this article, or claim that may be made by its manufacturer, is not guaranteed or endorsed by the publisher.
